# Assessing the Variability of Cell-Associated HIV DNA Quantification through a Multicenter Collaborative Study

**DOI:** 10.1128/spectrum.00243-22

**Published:** 2022-06-06

**Authors:** Yann Le Duff, Kathleen Gärtner, Eloise J. Busby, Annalisa Dalzini, Sivapragashini Danaviah, José Luis Jiménez Fuentes, Carlo Giaquinto, Jim F. Huggett, Matthew Hurley, Anne-Geneviève Marcellin, María Ángeles Muñoz-Fernández, Denise M. O’Sullivan, Deborah Persaud, Laura Powell, Peter Rigsby, Paolo Rossi, Anita de Rossi, Lilly Siems, Theresa Smit, Sarah A. Watters, Neil Almond, Eleni Nastouli

**Affiliations:** a Division of Infectious Disease Diagnostics, Centre for AIDS Reagent, National Institute for Biological Standards and Control, South Mimms, United Kingdom; b Department of Infection, Immunity and Inflammation, UCL Great Ormond Street Institute of Child Health, London, United Kingdom; c National Measurement Laboratory, LGC group Teddington, Middlesex, United Kingdom; d Section of Oncology and Immunology, Department of Surgery, Oncology and Gastroenterology, University of Padova, Padua, Italy; e Africa Health Research Institute, Durban, South Africa; f Instituto Investigación Sanitaria Gregorio Marañón, Laboratorio InmunoBiología Molecular and Spanish HIV HGM BioBank, Madrid, Spain; g Department for Woman’s and Child’s Health, University of Padova, Padua, Italy; h Sorbonne Université, INSERM, Institut Pierre Louis d’Epidémiologie et de Santé Publique (IPLESP), Assistance Publique-Hôpitaux de Paris (AP-HP), Pitié Salpêtrière Hospital, Department of Virology, Paris, France; i The Johns Hopkins University School of Medicine, Baltimore, Maryland, USA; j Department of Pediatrics, University of Rome Tor Vergata, Rome, Italy; k Immunology and Molecular Oncology Unit, Veneto Institute of Oncology IOV-IRCCS, Padua, Italy; l Division of Analytical Biological Sciences, National Institute for Biological Standards and Control, South Mimms, United Kingdom; Johns Hopkins Hospital

**Keywords:** collaborative study, diagnostics, HIV DNA, quantification variability, reference materials, human immunodeficiency virus

## Abstract

Reliable and accurate quantification of cell-associated HIV DNA (CA HIV DNA) is critical for early infant diagnosis, clinical management of patients under therapy, and to inform new therapeutics efficacy. The present study assessed the variability of CA HIV DNA quantification obtained from various assays and the value of using reference materials to help harmonize the measurements. Using a common set of reagents, our multicenter collaborative study highlights significant variability of CA HIV DNA quantification and lower limit of quantification across assays. The quantification of CA HIV DNA from a panel of infected PBMCs can be harmonized through cross-subtype normalization but assay calibration with the commonly used 8E5 cell line failed to reduce quantification variability between assays, demonstrating the requirement to thoroughly evaluate reference material candidates to help improve the comparability of CA HIV DNA diagnostic assay performance.

**IMPORTANCE** Despite a global effort, HIV remains a major public health burden with an estimated 1.5 million new infections occurring in 2020. HIV DNA is an important viral marker, and its monitoring plays a critical role in the fight against HIV: supporting diagnosis in infants and underpinning clinical management of patients under therapy. Our study demonstrates that HIV DNA measurement of the same samples can vary significantly from one laboratory to another, due to heterogeneity in the assay, protocol, and reagents used. We show that when carefully selected, reference materials can reduce measurement variability and harmonize HIV DNA quantification across laboratories, which will help contribute to improved diagnosis and clinical management of patients living with HIV.

## INTRODUCTION

Cell-associated (CA) human immunodeficiency virus (HIV) DNA is a clinically relevant and commonly monitored biomarker. Levels of CA HIV DNA have been shown to (i) predict disease progression, development to AIDS and HIV-associated neurological disorders (1–5), (ii) anticipate therapeutic failure and residual viremia upon treatment initiation (6–9), (iii) correlate with success of de-escalation therapy, and the magnitude and delay of viral rebound upon treatment interruption (1, 10–12). Because integrated CA HIV DNA durably persists in infected cells in the absence of viral RNA, it has also been widely used as an indicator of the size of the viral reservoir, particularly during clinical trials assessing new therapeutic strategies to eradicate latent HIV infection (13–15). In addition, CA HIV DNA is the target of choice for Early Infant Diagnosis (EID) since serology is not optimal due to the presence of passively transferred maternal antibodies in the infant and viral RNA detection may be masked as a result of peri-natal antiretroviral therapy (16). Since CA HIV DNA measurement informs clinical management of patients, robust quantification is paramount. Sensitive assays, consistently capable of detecting very low quantities of CA HIV DNA, are required, particularly in the context of EID and to assess the efficacy of new therapeutic approaches toward an HIV cure.

To quantify CA HIV DNA, total DNA is first extracted from clinical samples and specific HIV and cellular targets are then amplified, usually by quantitative real-time PCR (qPCR) or digital droplet PCR (dPCR). Over the years, some standardization of procedures used to detect HIV nucleic acids have emerged. Nevertheless, the performance and sensitivity of assays can be significantly impacted by parameters, including, the extraction method, the selection and number of viral or cellular targets, the sequence of the primers and probe as well as the overall workflow. In addition, interlaboratory and inter-operator inconsistencies have also been reported. One approach to tackle assay variability and harmonize results has been through the development of common reference materials to calibrate assays and underpin standardized protocols. The principles of biological standardization have been applied to calibrate assays and report the results of test samples normalized against the calibrant. The World Health Organization’s Expert Committee on Biological Standardization (WHO’s ECBS) has established international standards (ISs) to underpin nucleic acid tests (NATs) for most blood borne pathogens (17). The widespread availability and application of this primary calibrant by clinical laboratories and commercial assay manufacturers has not only increased data comparability between assays and laboratories but also demonstrated marked harmonization of measurement between assays produced by different manufacturers (18, 19).

In contrast with genomic HIV RNA, there is currently no WHO IS for the quantification of CA HIV DNA and scientists have been using their own preparation of plasmids encoding HIV genes, diluted in human genomic DNA, or DNA extracts from chronically infected cells bearing a proviral DNA copy, including the 8E5, U1, ACH2, and OM10.1 cells (20–25). However, there is uncertainty whether it is possible to compare the performance of assays calibrated using these different materials. We undertook a multicenter collaborative study involving laboratories which were expert in undertaking EID to assess the sensitivity and assay variability for CA HIV DNA quantification. The results generated by members of the EPIICAL consortium highlight the current degree of assay variability and demonstrate the benefit of developing common reference materials to help harmonize CA HIV DNA quantification. As a result of improved comparability of CA HIV DNA diagnostic assay performance and read-out, improvements in the clinical management of patients may be achieved.

## RESULTS

**FIG 1:**
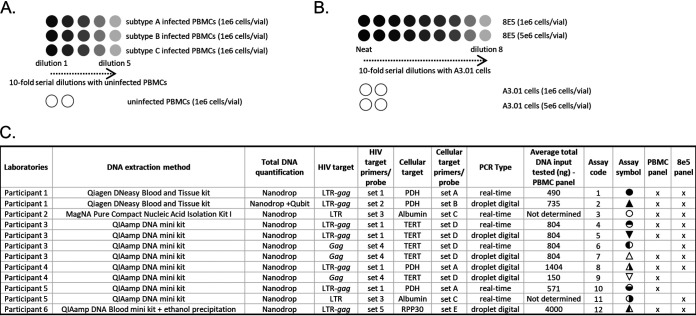
Study design and assay overview. (A) Panel consisting of PBMCs infected with HIV subtype A, B or C serially diluted with uninfected PBMCs as well as uninfected PBMCs only, as negative control (1e6 cells per vial). (B) Panel consisting of 8E5 cells serially diluted with A3.01 cells as well as A3.01 cells only, as negative controls (1e6 or 5e6 cells per vial). (C) Summary of assays performed by participating laboratories for the detection of either the PBMC panel and/or the 8E5 panel.

First, CA HIV DNA was quantified in a panel of PBMCs infected with HIV-1 subtype A, B or C (Fig. 1A). Assessment was conducted by 6 participating laboratories, which performed a total of 10 NATs, detecting multiple HIV and cellular targets, either by qPCR or dPCR (Fig. 1C). Upon receipt of the samples, the participants resuspended the pellet of PBMCs in PBS, carried out DNA extraction and quantified the total DNA amount by Nanodrop. Each vial contained 1e6 cells as determined using a hemocytometer during the preparation of the panel. Assuming that one human diploid cell contains ~6.5 pg of total DNA (26), the total amount of DNA in 1e6 cells is expected to be ~6.5 μg, or 1 μg per ~150,000 cells. Total amount of DNA recovered by the participants was similar between extraction methods and slightly below the expected target, with a median of 4.2 μg, except for assay 12, which included a post extraction precipitation step, and recovered double the amount of DNA (Fig. S1A). Because CA HIV DNA is reported as a ratio of HIV DNA copies per 1e6 cells, we then assessed the number of cells per vial quantified by PCR using a reference gene (Fig. S1B). The total quantity of PBMCs per vial reported by the participants was below the anticipated 1e6, with a median of 0.43× 1e6 cells. Assays 9 and 10 detected significantly more cells per vial and per μg of extracted DNA compared to the expected 150,000 cells (Fig. S1C) which is likely due to a bias in the quantification of the cellular target. The remaining assays measured a quantity of PBMCs per μg of DNA slightly lower than expected which could be attributed to an overestimation of total DNA concentration with Nanodrop as previously reported (27, 28). A particularly low quantity of cells per μg of DNA was quantified with assay 12, that could be caused by an inaccurate quantification of total DNA concentration.

**FIG 2:**
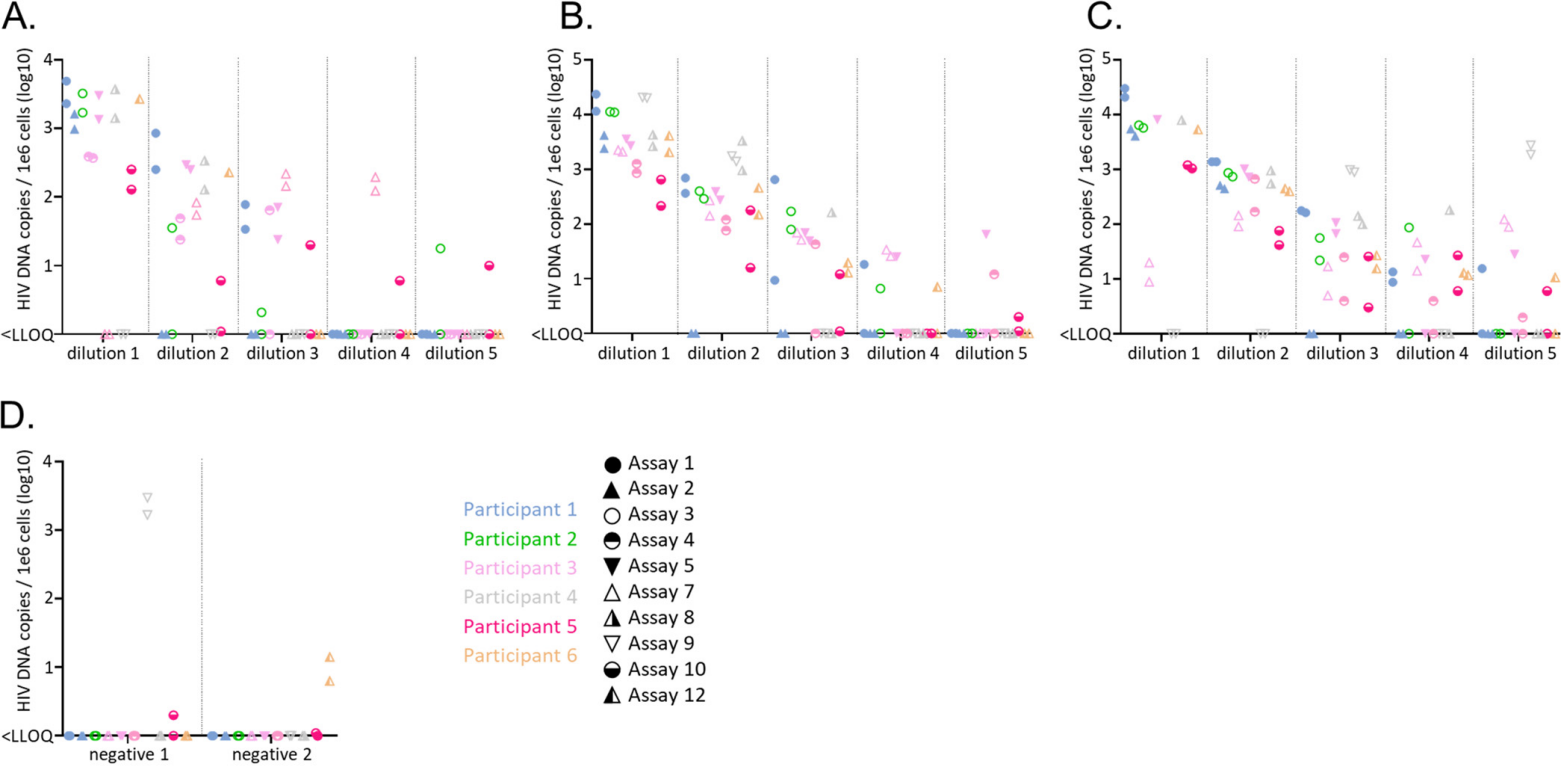
Quantification of CA HIV DNA in the panel of PBMCs. Quantifiable measurements of CA HIV DNA in the panel of PBMCs infected with HIV subtype A (A), subtype B (B), subtype C (C), or uninfected (D) were reported for 10 assays used by the 6 study participants. For each subtype, five 10-fold dilutions were tested as well as two uninfected controls. Two quantifications are reported for each subtype and dilution (when available), corresponding to the two identical panels assessed by each participant. The participants are color coded and the assays identified by a unique icon. qPCR are represented as circles, dPCR as triangles.

Second, CA HIV DNA quantification was determined in the two duplicate panels of PBMCs (Fig. 2). The quantities reported by the participants varied significantly with 2 to 2.5 logs difference frequently observed between assays, across the 3 subtypes. The inter-assay variability increased as the HIV positive PBMCs were diluted. This correlates with an assay dependent decrease in the reproducibility of the quantification between duplicates as the CA HIV DNA concentration declines (Fig. S2). Assays 7 and 9 targeting HIV-1 *gag* were designed to detect subtype B and therefore showed inconsistent results for subtype A and C due to the presence of several mismatches between the primers, probe and the sequence of the HIV isolates used to create the panel (data not shown). Marked improvements were observed when using an assay targeting the LTR region. Overall, the best consensus between assays was observed for subtype B, followed by subtype A and subtype C, primarily based on detection and quantification disparities for low CA HIV DNA concentrations. Out of the 40 vials of uninfected PBMCs, 6 were reported positive, likely due to cross contaminations (Fig. 2D).

**FIG 3:**
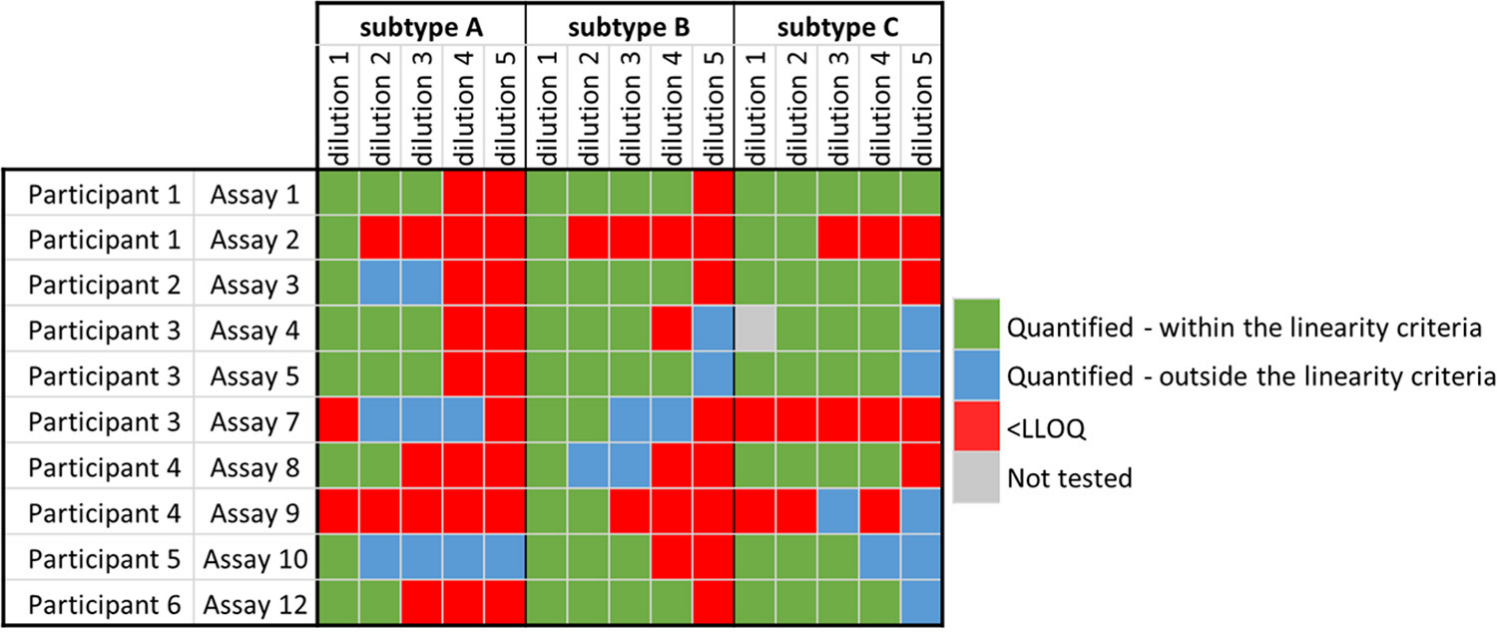
Evaluation of the limit of quantification of each assay. Quantifications, which followed the expected 10-fold dilutions, were selected and highlighted in green. Quantifications that did not meet the linearity criteria are depicted in blue. Samples that were found below the lower limit of quantification are depicted in red. One sample not tested is depicted in grey.

Quantification of CA HIV DNA in the panel of PBMCs is expected to follow a 10-fold decrease profile as the target has been 10-times serially diluted (Fig. 1A). Therefore, to assess the accuracy of the measurements reported by the participants, particularly for low concentrations of targets, and to facilitate comparison of the lower limit of quantification (LLOQ) between assays, an assessment of data that met the dilutional linearity criteria (details in M&M) was performed (Fig. 3). The proportion of reported positive results meeting the linearity criteria (highlighted in green) therefore indicated the robustness of the assay. Interestingly, several data points did not satisfy the linearity criteria for low CA HIV DNA quantities, as illustrated by dilution 5 in subtype C. This observation emphasizes the challenge of accurately measuring very low quantity of CA HIV DNA and suggests on overestimation of the LLOQ for some assays. Even when the linearity criteria are satisfied, the LLOQ varied by up to 3 logs between assays and across subtypes which correlated with the overall variability highlighted in Fig. 2. Assay 1 performed by participant 1 was the most robust overall, consistently detecting the lowest concentration of CA HIV DNA of all assays and across subtypes. In addition, all quantifications met the linearity criteria. These results were however not reproduced by participant 3 and 5 using very similar assays (assays 4 and 10, respectively), illustrating operator to operator variability.

**FIG 4:**
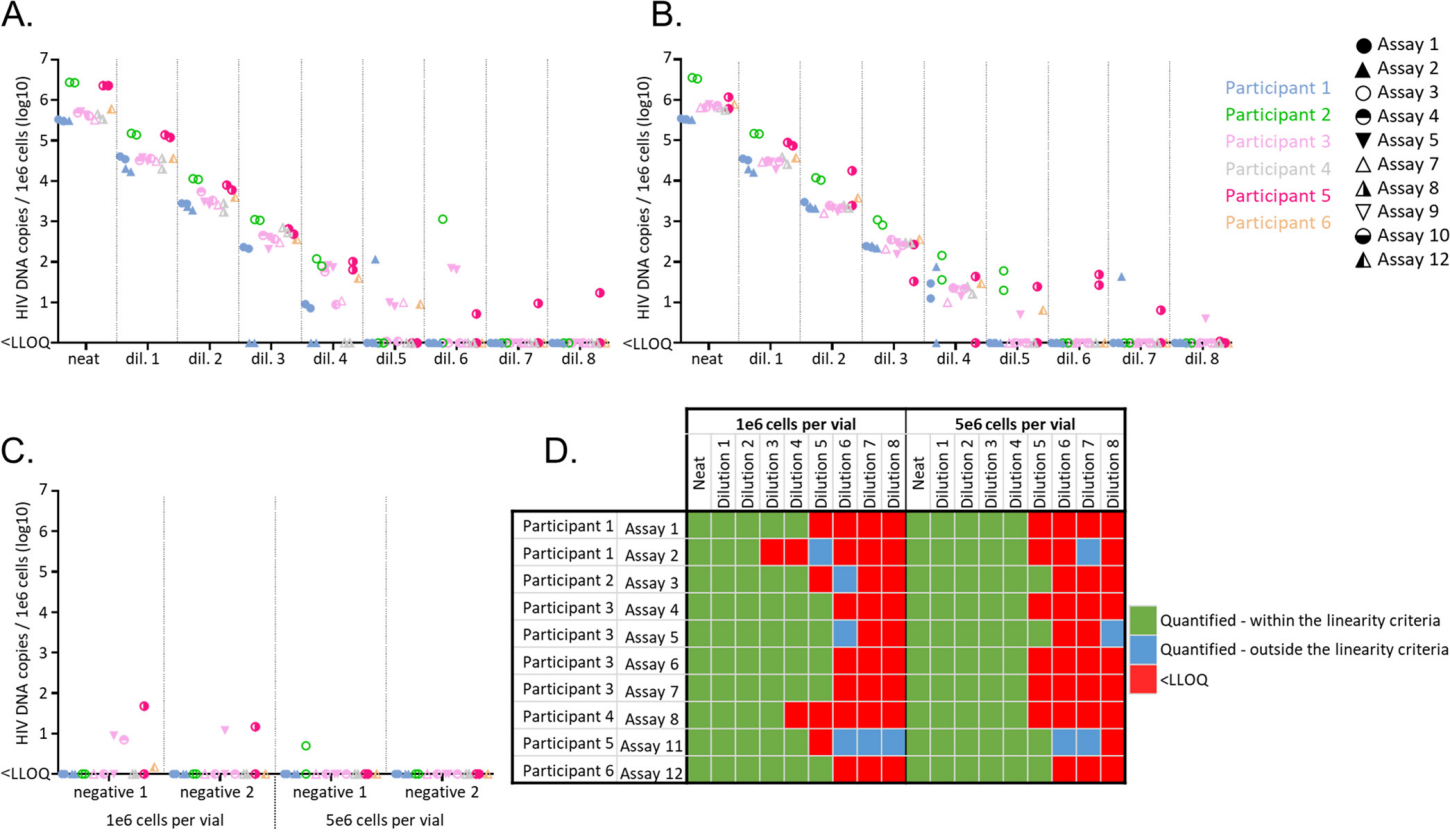
Quantification of CA HIV DNA in the panel of 8E5 cells. Quantifiable measurements of CA HIV DNA in the panel of 8E5 cells containing either 1e6 cells per vial (A) or 5e6 cells per vial (B) and in the panel of A3.01 only (negative control) (C), were reported for 10 assays used by the 6 study participants. For each set, the neat sample and eight 10-fold dilutions were tested, as well as two samples containing A3.01 cells only (negative controls). Two replicates are reported for each set and dilution, corresponding to the two identical panels assessed by the participants. The participants are color coded and the assays identified by a unique icon. qPCR are represented as circles, dPCR as triangles. (D) Quantifications which followed the expected 10-fold dilutions, were selected and highlighted in green. Positive results, which did not meet the linearity criteria, are depicted in blue. Samples that were found below the lower limit of quantification are depicted in red.

Next, CA HIV DNA was quantified in a panel of 8E5 cells which is commonly used as a reference reagent for HIV DNA measurements (20, 29, 30) (Fig. 1B, Fig. 4). Relatively high concordance in the quantification was observed up to dilution 3 at which point some assays failed to detect either one or the two duplicate vials. None of the quantifications reported for dilution 6 met the linearity criteria (Fig. 4D) due to level of methodological sensitivity which in cases was due to false-positive results caused by cross contamination. This was also suspected for signals found in dilutions 7, 8 and in the A3.01 cells only, mainly in the set containing 1e6 cells per vial (Fig. 4C). For quantifications that met the linearity criteria, the maximum difference in the LLOQ between assays was 3 logs for the panel containing 1e6 cells per vial, due to underperformance of the dPCR assays 2 and 8, used by participant 1 and 4, respectively (Fig. 4D). The LLOQ of these 2 assays as well as assay 3 and 11 was improved when using the panel containing 5e6 cells per vial. In contrast, the LLOQ was unaltered for assays 1, 5 and 12 and increased for assay 4, 6, and 7. Overall, the best performance was obtained with assay 12 performed by participant 6, with reliable detection up to dilution 5 for both sets of 8E5 cells.

**FIG 5:**
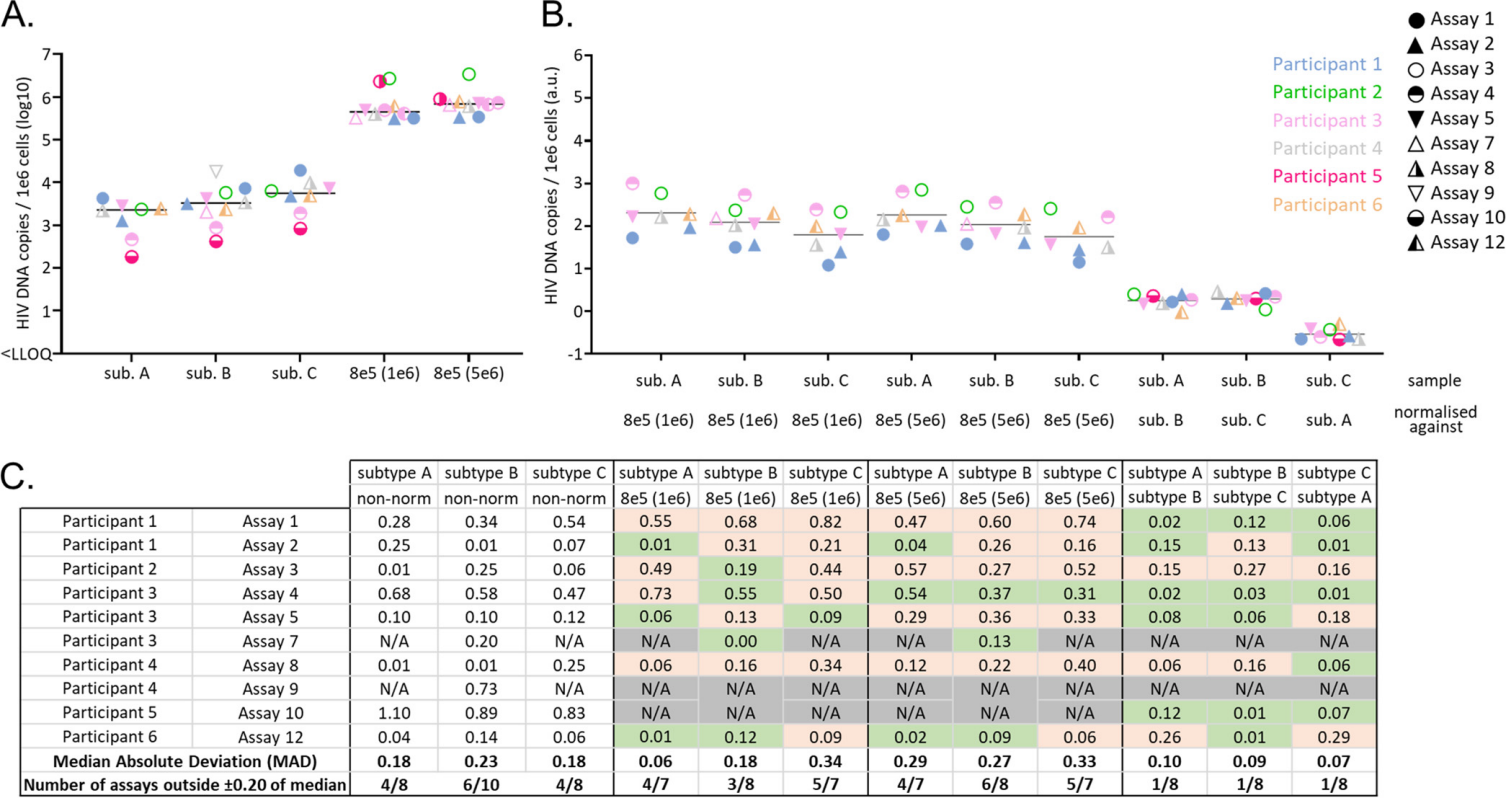
Effect of normalization of CA HIV DNA quantification in PBMCs. (A) Mean of nonnormalized dilution-factored CA HIV DNA quantification selected with the defined linearity criteria, as well as the median between assays are represented. (B) Normalization of the PBMC panel quantification with data obtained from the 8E5 cell panel (1e5 and 5e6 cells per vial) as well as cross-subtype normalization. The participants are color coded and the assays identified by a unique icon. qPCR is represented as circles, dPCR as triangles. (C) For nonnormalized and normalized data, the distance to the median, median absolute deviation, and the number of assays falling outside 0.20 of median are reported. Reduction of the distance to the median after normalization is highlighted in green and increase in orange. NA indicates that the calculation was not possible.

In order to further simplify the overall comparison of accurate quantifications between assays, the arithmetic mean of all measurements that met the linearity requirements was calculated, factoring in the dilutions (Fig. 5A). Measurements outside the linearity criteria were excluded to prevent the introduction of a bias in the calculation. This approach allowed to display each assay with one representative quantification per subtype and set of 8E5 cells. As expected, excluding data failing the linearity criteria reduced the quantification variability presented in Fig. 2 to around 1.5 to 2 logs for the panel of PBMCs. Interestingly, assay 4 and 10 consistently detected lower CA HIV DNA than the other assays, across the 3 subtypes of this panel. This discrepancy was partially explained for assay 10 by a general overestimation of the cellular target (Fig. S3).

Finally, we evaluated whether the use of common reference reagent helped reduce the variability in the measurements of CA HIV DNA in the PBMC panel. As illustrated in fig. 5B and C, quantification in the PBMCs panel across subtypes was not significantly harmonized between assays when normalizing against the data obtained from the panel containing 1e6 or 5e6 8E5 cells. In contrast, cross-subtype normalization of the PBMC panel quantification significantly decreased the median absolute deviation and the proportion of assays inside +/−0.20 log of the median, with only one participant per subtype falling outside this criterion after normalization.

## DISCUSSION

This study highlights significant inter-assay variability in the quantification of CA HIV DNA. Variability is exacerbated for samples containing low quantities of analyte, which are found either accurately quantified, inaccurately quantified, or negative, leading to significant differences in the LLOQ between assays. Increasing the quantity of total DNA tested, either per PCR or through multiple replicates, is expected to improve quantification reliability for low target quantities, and consequently assay LLOQ. In the present study, this seems to particularly apply to dPCR. First, the LLOQ of assays 2 and 8 was improved with higher concentration of 8E5 cells and the underperformance of assay 2 for the PBMC panel was likely caused by a lower quantity of cell equivalent per reaction used in our study compared to the one necessary for optimal performance of the assay (31). Second, assay 12, which quantified CA HIV DNA in the PBMC panel using the highest total DNA quantity, 4 μg, was one of the most sensitive assays. Third, this is also consistent with a poor LLOQ of assay 9 when detecting the PBMC subtype B samples, which was performed with an input of only 150 ng of DNA. Interestingly, high input quantity, however, does not seem necessarily required to achieve robust quantification of low quantity targets with qPCR, as demonstrated by results obtained with assay 1 and to a smaller degree assay 4. The fluctuation in the frequency of molecular dropout, where the target is present in the PCR but is not detected, is another factor that could contribute to inter-assay variability in the LLOQ, as previously reported (32, 33). Our results also highlight variability in the quantification of high concentrations of CA HIV DNA as presented in Fig. 2 and Fig. 5A, which is illustrated by a constant under quantification by assay 4 and 10, across the 3 subtypes of the PBMC panel. This is partially explained for assay 10 by a general overestimation of the quantification of cellular target which highlights the often-overlooked requirement of a robust and reproducible quantification of cellular target for accurate CA HIV DNA measurement (Fig. S3). The assay itself is however probably not the root cause of this discrepancy but rather operator to operator variability as assay 10 and 1 targeted HIV (LTR) and the cellular gene (PDH) with identical primers and probe, and follow the overall same standard operating procedure.

Indeterminate PCR results, defined as inconclusive tests, are frequently observed and remain one of the main challenges of EID (34–38). As national programs aiming at preventing mother-to-child transmission are successfully implemented, the positive predictive value of a single diagnostic test will continue to decrease. This highlights the requirement of using appropriate controls in laboratories performing these tests and that confirmatory testing is performed to manage inconclusive results, as recommended by the WHO (39). A recent report showed that 76% of samples returning an inconclusive result are found negative upon repeated testing suggesting that most indeterminate PCR are false positive (40).

In our study, false-positive results are occasionally reported for the panel of uninfected PBMCs and A3.01 cells. It is extremely unlikely that these results were caused by contaminations of the reagents shared with the participants as the panel of uninfected PBMCs and A3.01 cells were prepared following rigorous measures to prevent cross contamination and positive signals would have been more widely reported in the negative control samples. Our study demonstrates that false-positive results is a laboratory specific technical artifact. False positive results are likely to occur as a consequence of cross contamination (either from positive samples or amplicon) during the preparation of the PCR or during the DNA extraction step, particularly when manual processing is performed. While there are discussions in the literature about threshold setting (41–44) or droplet size variation (45–47) exacerbating the issue, false positives are a risk for any PCR format due to the exquisite sensitivity afforded by this detection method. Assays used for EID not only require high sensitivity but also high specificity in the detection of trace amount of target (<10 copies per reaction) because false-positive results are clinically impactful. In addition to repeat testing recommended by international guidelines and the implementation of quality assurance programs (39), the inclusion of appropriate controls to monitor the diagnostic process, such as blank extraction sample and nontemplate control is critical to help manage false-positive results and enable the high analytical sensitivity required to make these clinical measurements. These challenges will be further addressed during the collaborative study to assess WHO IS candidates where a larger set of data will help minimize the effects of confounding factors.

Subtype A and C are the most prevalent subtypes in the African continent, where implementation of robust and reliable EID is particularly critical and were therefore included in our study. Because the initial quantity of CA HIV DNA may not be identical in the 3 subtypes tested, the PBMC panel was used to compare the efficiency of assays within a subtype and not to compare the performance of each individual assay between subtypes. However, variations in the quantification hierarchy between assays across subtypes could indicate a sequence dependent effect on the measurements. As shown in Fig. 5A, such variation is small and even if we cannot completely rule out an impact of the HIV target sequence on quantification, this factor seems to be minimal.

The use of common reference materials has been shown to increase data harmonization for the quantification of various analytes, including viral genomes (48, 49). Because the first HIV NATs have been developed over 30 years ago and have continuously improved since, it is important to recognize that the quantification variability observed in the present study is not as severe as the one observed for the detection of other pathogens, including newly emerging viruses (48, 49). Interestingly, calibrating the assay against the 8E5 cells, one of the most commonly used reagents to quantify CA HIV DNA, fails to harmonize the data obtained with the PBMC panel. One explanation could lie in the heterogenicity of the CA HIV DNA population between the 8E5 cell line and the infected PBMCs used in our study. Whereas the 8E5 cell line contains integrated proviral double-stranded DNA, the PBMCs probably contain various other CA HIV DNA species, including episomal DNA, either as linear or circular forms, which might be detected differently depending on the assay used. Another hypothesis is that chromosomal modifications or duplications may have occurred during numerous cell passages, from the original CEM, A3.01, to the 8E5 cell line, resulting in altered number of cellular targets compared to PBMCs, therefore introducing a bias in the quantification of the number of cells. Finally, as mentioned above, we cannot exclude that slight genetic variations between the sequence of the HXB2 provirus integrated in the 8E5 cell line and the isolates used to produce the panel of PBMCs impacted the assay detection. The quantity of CA HIV DNA in the 8E5 cell line has been shown to vary drastically depending on the batch (31). Our study highlights another limitation of using this cell line as reference to quantify CA HIV DNA in infected PBMCs. Importantly, for the panel of PBMCs, cross-subtype normalization significantly reduces both the median absolute deviation and the number of assays outside 0.20 of the medians which is particularly exemplified by a drastic correction of a constant quantification bias for assay 4 and 10. These results have important implications for the design of reference materials, suggesting that individual standard for each HIV-1 subtype may not be required and that one IS would be able to harmonize data from calibrated assays detecting various subtypes. A critical feature of a suitable IS is commutability, which means that the measurement from the standard mirrors the measurement from a clinical sample. To assess commutability, direct evaluation of how the standard behaves compared to clinical samples in different assays and in different matrices is determinant. For example, it has been reported that a variable bias was observed when the Abbott RealTime CMV assay was calibrated using the human cytomegalovirus WHO IS NAT resuspended in whole-blood compared to plasma (50). CA HIV DNA is detected from various matrices, including purified PBMCs, purified CD4^+^ T-cells, whole blood and dry blood spot. It is therefore vital that a broad selection of assays using various matrices are carefully assessed during the collaborative study to evaluate WHO IS candidates.

### Conclusion.

The present study highlights variability between various assays in the quantification of CA HIV DNA in infected PBMCs. Unreliable results are frequently reported for very low concentrations of CA HIV DNA, consistent with indeterminate PCR observed for EID. Variability in the quantification of higher concentrations of CA HIV DNA is also observed, though to a lower degree. Consistent variability patterns across subtypes allowed to harmonize data through cross-subtype normalization but calibration of assays with the 8E5 cells failed to do so. The findings presented in our study rely on a limited set of data and additional investigation with stronger statistical power is required, particularly to further assess the commutability of various reference material candidates to harmonize data obtained from clinical samples. This will be addressed through a larger collaborative study following the recent endorsement by the WHO to develop the first IS for the quantification of CA HIV DNA. This calibrant will be instrumental to increase comparability in CA HIV DNA quantifications to help improve the diagnosis and clinical management of HIV infected patients.

## MATERIALS AND METHODS

### Participating laboratories.

EPIICAL is a large international consortium aiming to implement novel strategies to obtain long-term viral remission in early treated HIV infected children. These include broadly neutralizing antibodies and therapeutic vaccination and as defining virological endpoints is key for successful clinical trials, standardizing potential virus markers is essential. Six laboratories in Europe and the US are partners in the consortium and have contributed to this analysis.

### Study design.

A total of 12 assays designed to detect CA HIV DNA for EID were performed by 6 clinical laboratories part of the EPIICAL consortium (Fig. 1). To assess the ability of the participants to quantify CA HIV DNA, two panels of samples were produced at NIBSC. The first panel consisted of PBMCs infected with HIV-1 subtype A, B or C and 10-fold serially diluted with uninfected PBMCs. For each of the 3 subtypes, 5 dilutions were selected following the results of a short pilot study (data not shown) so that assays would reach an endpoint in order to determine their LLOQ. Two vials of uninfected PBMCs were also included in the panel as negative controls. The samples were provided as dry pellet of 1e6 cells per vial. The second panel consisted of 8E5 cells 10-fold serially diluted with parental A3.01 cells. Neat 8E5 cells and eight 10-fold serial dilutions were prepared and provided as dry pellet containing 1e6 or 5e6 cells per vial. Each participant received 2 blinded panels of PBMCs and 8E5 cells. Shipment of the reagents from NIBSC to all the participants was performed under the ISO9001 quality system. The participants were asked to resuspend the pellet of cells in PBS, perform nucleic acid extraction and determine the CA HIV DNA copy number per 1e6 cells using one or more NATs of their choice. A MIQE table reporting comprehensive information on DNA extraction and PCR amplification protocols are included in Table S1.

### Preparation of the panels of PBMC and 8E5 cells.

Buffy coats from healthy individuals were obtained from the NHS blood transfusion center. The buffy coats were diluted 1:2 with Ca^2+^/Mg^2+^-free PBS, layered on top of Ficoll Paque Plus (GE Healthcare cat. number 17-1440-02) then centrifuged at 500 × *g* for 30 min at room temperature with deceleration without brake. The layer of PBMCs was isolated and washed twice in 5 volumes of Ca^2+^/Mg^2+^-free PBS. The cells were frozen in 90% Fetal Bovine Serum (PAN-Biotech cat. number FB-1001) plus 10% DMSO (Sigma cat. number D8418). Freshly thawed PBMCs were grown for 3 days in RPMI (Sigma cat. number R0883) supplemented with Fetal Bovine Serum (10% vol/vol, PAN-Biotech cat. number FB-1001), glutamine (Sigma cat. number G7513), penicillin/streptomycin (Sigma cat. number P0781) and 5 μg/mL of PHA (Sigma cat. number L1668). On the day of infection, PBMCs were washed twice with PBS and resuspended in medium without PHA but containing 20 IU/mL of IL-2 (CFAR cat. number 0901). The cells were inoculated with one HIV primary isolate from subtype A, B, or C at an MOI = 0.01 (CFAR cat. number 1089, 1196, 1080, respectively), for which near full-length sequence was available (GenBank accession numbers FJ670519, 1KT276256, EU884500.1, respectively). Polybrene (Sigma cat. number 9868) was added to a final concentration of 2 μg/mL and the cells were centrifuged at 1200 × *g*, 2h, at room temperature. After incubation overnight at 37°C, the cells were diluted with eight volumes of IL-2 containing medium and incubated for a further 24h at 37°C. Forty-eight hours postinoculation, infected PBMCs were counted twice using an hemocytometer and 10-times serially diluted with uninfected PBMCs. For each dilution, 1e6 cells were centrifuged, washed once with PBS and the supernatant was removed and the cell pellet air-dried for 5 min and frozen at –80°C. Uninfected PBMC used as negative controls were prepared the same way but at separate days to avoid any cross-contamination with infected PBMCs.

The 8E5 cells (kindly provided by Dr. Kathleen Gärtner) and parental A3.01 cells (CFAR cat. number 0098) were grown in RPMI (Sigma cat. number R0883) supplemented with Fetal Bovine Serum (10% vol/vol, PAN-Biotech cat. number FB-1001), glutamine (Sigma cat. number G7513), and penicillin/streptomycin (Sigma cat. number P0781). Five days post recovery, the 8E5 and A3.01 cells were counted and the 8E5 cells were 10-times serially diluted with A3.01. For each dilution, 1e6 and 5e6 cells were centrifuged, washed once with PBS, and the supernatant was removed and the cell pellet air-dried for 5 min and frozen at –80°C. A3.01 cells used as negative controls were prepared in the same way but at separate days to avoid any cross-contamination with 8E5 cells.

### Data reporting and analysis.

The data presented in this study are raw quantification of CA HIV DNA reported by the participants as quantifiable signal in CA HIV DNA copies per 1e6 cells. To generate fig. 3 and 4D, a linear regression of log copy number against log dilution was performed for each assay. The data were used for further analysis and highlighted in green if the following linearity criteria were met: coefficient of determination exceeding 0.9; slope between −1.2 and −0.8 (expected value of −1.0 for 10-fold dilutional linearity). Where any high dilutions (low CA HIV DNA concentrations) caused a participant to fail these criteria, the quantification for that dilution was highlighted in blue and excluded from further analysis as outside the linear range in this study.
