# Supplementary material for: Assessing the Variability of Cell-Associated HIV DNA Quantification through a Multicenter Collaborative Study
Source: Microbiol Spectr. 2022 Jun 6;10(3):e00243-22. doi: 10.1128/spectrum.00243-22 (PMC9241949; doi:10.1128/spectrum.00243-22)
Supplement: SUPPLEMENTAL FILE 2 — Supplemental material. Download spectrum.00243-22-s0002.pdf, PDF file, 0.4 MB [file spectrum.00243-22-s0002.pdf]

Figure S1

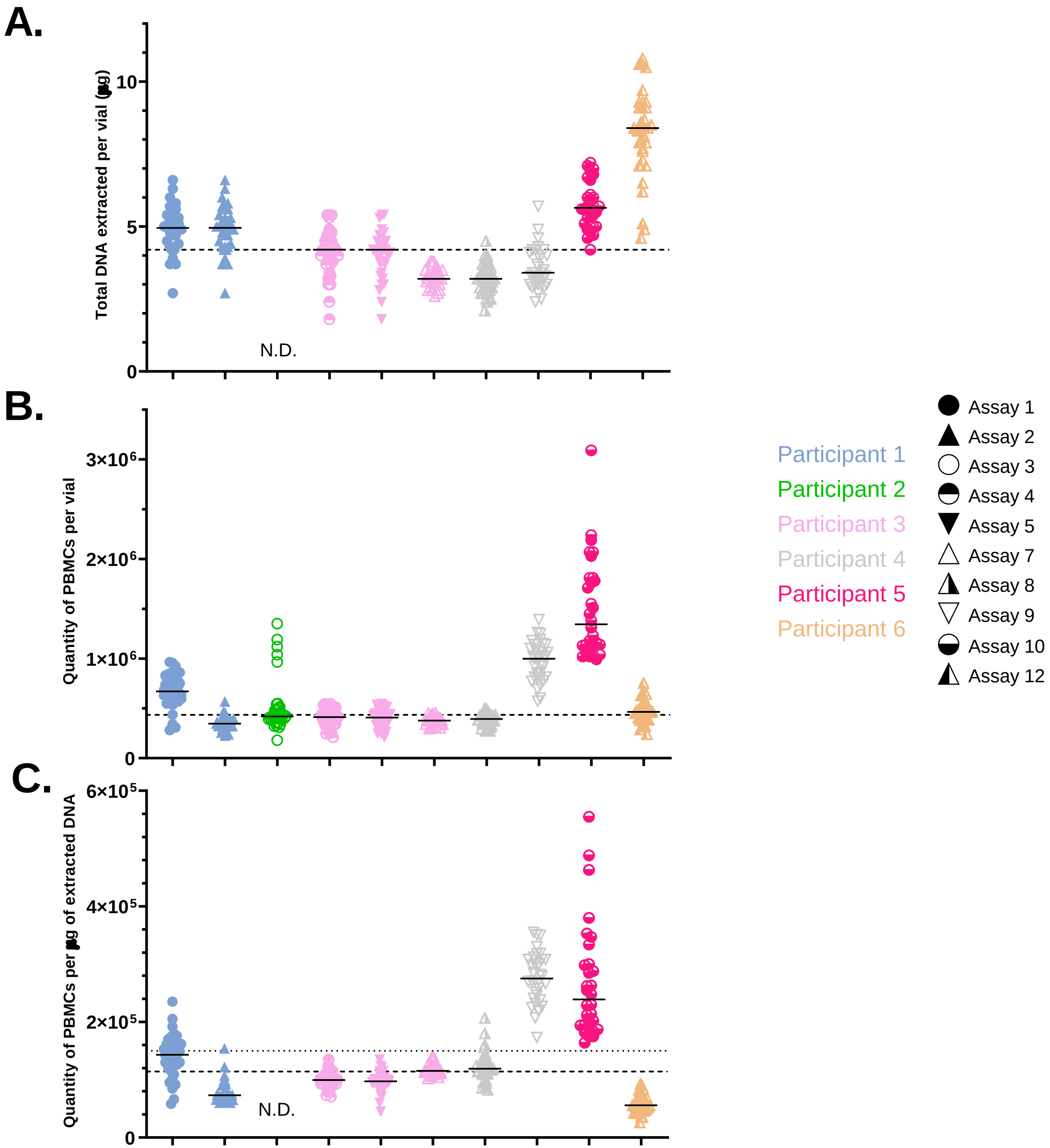

Figure S1. DNA extraction and quantification of cellular genes

(**A**) Total DNA quantity measured by Nanodrop in each vial received by the participants. The median for each participant is depicted and the grand median is indicated as a dashed line. N.D.: not determined (**B**) Total quantity of PBMCs per vial as calculated using PCR, targeting a cellular reference gene. The median for each participant is depicted and the grand median is indicated as a dashed line. (**C**) Quantity of PBMCs per µg of DNA extracted. The median for each participant is depicted and the grand median is indicated as a dashed line. 150,000 cells per µg of DNA, corresponding to 6.5pg of DNA per diploid cell, is indicated as a dotted line. N.D.: not determined. The participants are colour coded and the assays identified by a unique icon. qPCR are represented as circles, dPCR as triangles.

Figure S2

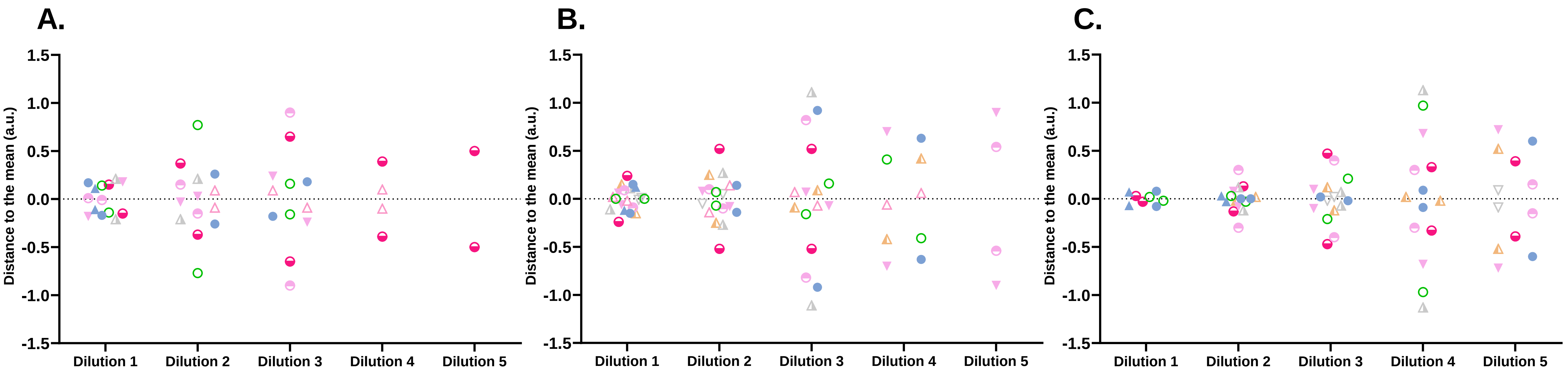

D.

		Average distance to the mean (a.u.)			
		Subtype A	Subtype B	Subtype C	**Median**
Participant 1	Assay 1	0.18	0.39	0.08	**0.18**
Participant 1	Assay 2	0.11	0.12	0.05	**0.11**
Participant 2	Assay 3	0.16	0.12	0.12	**0.12**
Participant 3	Assay 4	0.15	0.10	0.30	**0.15**
Participant 3	Assay 5	0.18	0.08	0.39	**0.18**
Participant 3	Assay 7	0.09	0.06	N/A	**0.08**
Participant 4	Assay 8	0.21	0.27	0.12	**0.21**
Participant 4	Assay 9	N/A	0.03	0.06	**0.05**
Participant 5	Assay 10	0.39	0.38	0.33	**0.38**
Participant 6	Assay 12	N/A	0.20	0.07	**0.14**

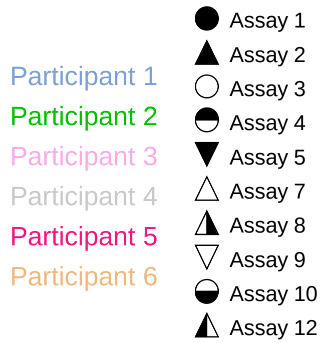

Figure S2. Variability in the quantification of the two replicate panels of PBMCs.

The difference to the median of each replicate quantification calculated for each dilution is depicted for subtype A (**A**), subtype B (**B**), and subtype C (**C**). The participants are colour coded and the assays identified by a unique icon. qPCR are represented as circles, dPCR as triangles. (**D**) Table summarising the average distance to the mean for each assay.

Figure S3

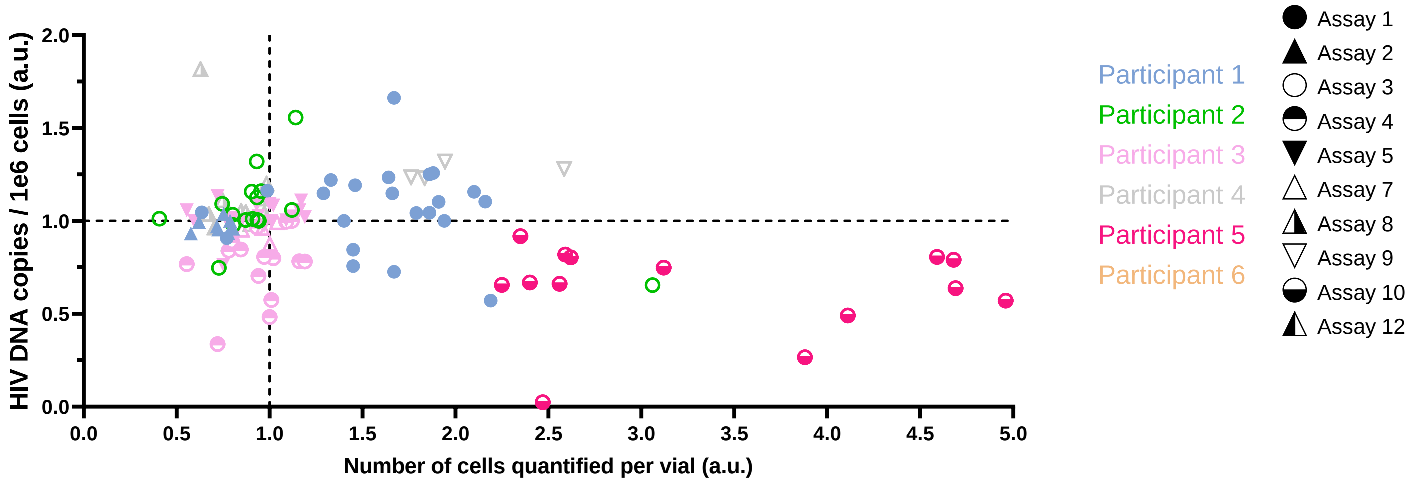

Figure S3. Evaluation of the impact of the cellular target quantification on the CA HIV DNA copies per million cells.

Normalised representation of the impact of the quantification of cellular reference target on CA HIV DNA quantification, reported in copies per million cells. The participants are colour coded and the assays identified by a unique icon. qPCR are represented as circles, dPCR as triangles.
